# Organoids as a biomarker for personalized treatment in metastatic colorectal cancer: drug screen optimization and correlation with patient response

**DOI:** 10.1186/s13046-024-02980-6

**Published:** 2024-02-27

**Authors:** Lidwien P. Smabers, Emerens Wensink, Carla S. Verissimo, Esmee Koedoot, Katerina-Chara Pitsa, Maarten A. Huismans, Celia Higuera Barón, Mayke Doorn, Liselot B. Valkenburg-van Iersel, Geert A. Cirkel, Anneta Brousali, René Overmeer, Miriam Koopman, Manon N. Braat, Bas Penning de Vries, Sjoerd G. Elias, Robert G. Vries, Onno Kranenburg, Sylvia F. Boj, Jeanine M. Roodhart

**Affiliations:** 1 Department of Medical Oncology, University Medical Center Utrecht (UMCU), Utrecht, The Netherlands; 2 HUB Organoids B.V, Utrecht, The Netherlands; 3 Molecular Cancer Research, Center for Molecular Medicine, UMCU, Utrecht, The Netherlands; 4 Department of Medical Oncology, Maastricht University Medical Center, Maastricht, The Netherlands; 5 Department of Medical Oncology, Meander Medical Center, Amersfoort, The Netherlands; 6 Present Address: Utrecht Platform for Organoid Technology (UPORT), UMCU, Utrecht, The Netherlands; 7 Department of Radiology, UMCU, Utrecht, The Netherlands; 8 Department of Epidemiology, Julius Center for Health Sciences and Primary Care, UMCU, Utrecht, The Netherlands; 9 Laboratory of Translational Oncology, Division of Imaging and Cancer, UMCU, Utrecht, The Netherlands

**Keywords:** Organoids, Oncology, Cancer, Precision medicine, Colorectal cancer, Chemotherapy, Drug screening

## Abstract

**Background:**

The inability to predict treatment response of colorectal cancer patients results in unnecessary toxicity, decreased efficacy and survival. Response testing on patient-derived organoids (PDOs) is a promising biomarker for treatment efficacy. The aim of this study is to optimize PDO drug screening methods for correlation with patient response and explore the potential to predict responses to standard chemotherapies.

**Methods:**

We optimized drug screen methods on 5–11 PDOs per condition of the complete set of 23 PDOs from patients treated for metastatic colorectal cancer (mCRC). PDOs were exposed to 5-fluorouracil (5-FU), irinotecan- and oxaliplatin-based chemotherapy. We compared medium with and without N-acetylcysteine (NAC), different readouts and different combination treatment set-ups to capture the strongest association with patient response. We expanded the screens using the optimized methods for all PDOs. Organoid sensitivity was correlated to the patient’s response, determined by % change in the size of target lesions. We assessed organoid sensitivity in relation to prior exposure to chemotherapy, mutational status and sidedness.

**Results:**

Drug screen optimization involved excluding N-acetylcysteine from the medium and biphasic curve fitting for 5-FU & oxaliplatin combination screens. CellTiter-Glo measurements were comparable with CyQUANT and did not affect the correlation with patient response. Furthermore, the correlation improved with application of growth rate metrics, when 5-FU & oxaliplatin was screened in a ratio, and 5-FU & SN-38 using a fixed dose of SN-38. Area under the curve was the most robust drug response curve metric. After optimization, organoid and patient response showed a correlation coefficient of 0.58 for 5-FU (*n* = 6, 95% CI -0.44,0.95), 0.61 for irinotecan- (*n* = 10, 95% CI -0.03,0.90) and 0.60 for oxaliplatin-based chemotherapy (*n* = 11, 95% CI -0.01,0.88). Median progression-free survival of patients with resistant PDOs to oxaliplatin-based chemotherapy was significantly shorter than sensitive PDOs (3.3 vs 10.9 months, *p* = 0.007). Increased resistance to 5-FU in patients with prior exposure to 5-FU/capecitabine was adequately reflected in PDOs (*p* = 0.003).

**Conclusions:**

Our study emphasizes the critical impact of the screening methods for determining correlation between PDO drug screens and mCRC patient outcomes. Our 5-step optimization strategy provides a basis for future research on the clinical utility of PDO screens.

**Supplementary Information:**

The online version contains supplementary material available at 10.1186/s13046-024-02980-6.

## Introduction

Therapeutic options for the treatment of patients with metastatic colorectal cancer (mCRC) are expanding rapidly, but heterogeneity in treatment responses, ranging from 10 to 90%, remains a fundamental challenge [1–4]. Predicting to which treatment a patient will respond is one of the holy grails in cancer research for enabling personalized care and improving patient survival. Despite considerable research in the field of predictive biomarkers, with the main focus on genetic biomarkers, there are clinically limited means to predict treatment efficacy. Genetic biomarkers do not predict response to chemotherapy, the cornerstone of treatment of mCRC. Additionally, genetic biomarkers like *RAS* and *BRAF* mutational status do not robustly predict treatment response for targeted treatments [5]. As a result, many patients receive ineffective, costly drugs and suffer unnecessary toxicities.

A promising predictive biomarker is in vitro response testing using patient-derived organoids (PDOs) [6, 7]. PDOs are (cancer) stem-cell derived, 3D self-organizing and proliferating structures comprised of epithelial cells representing their corresponding tumour genomically and phenotypically and are well suited for (high throughput) drug screening [7–11]. This makes PDOs a novel promising biomarker to predict treatment response for various cancer and treatment types, and a valuable screening platform to identify new treatment types [7, 12, 13]. Previous studies involving colorectal cancer (CRC) patients have demonstrated a correlation between organoid response and patient response to different forms of chemotherapy and targeted treatment [7, 14–22]. However, these results are not consistent, especially not for oxaliplatin which is one of the main treatments for mCRC [20, 23]. Variation between studies arise by using different combination screen set-ups, different response evaluation readouts and differences in components used in the screening medium [6, 7, 20, 24–26]. Especially the use of N-acetyl cysteine (NAC) in organoid culture media is point of debate [11]. NAC is known to interfere with platinum-based chemotherapy [27, 28] and notably studies that used NAC in oxaliplatin screening found no correlation between organoid sensitivity and patient response [20, 23]. This signals the need for first improving and standardizing drug screening methodologies before PDO screens can reliably be prospectively tested as biomarker in the clinic.

In this study, we aim to optimize the PDO drug screening methods with regards to screening medium, combination screen set-up, drug screen readout, curve fitting and drug response curve metrics. Subsequently, we explore if these optimized PDO screens can adequately predict treatment response in patients for standard-of-care systemic chemotherapies 5-FU/capecitabine, irinotecan- and oxaliplatin-based combination treatment. In addition, we investigate whether clinical factors, like mutational status, primary tumour location (sidedness) and prior chemotherapy treatment affect organoid sensitivity.

## Methods

### Study design

**Fig. 1:**
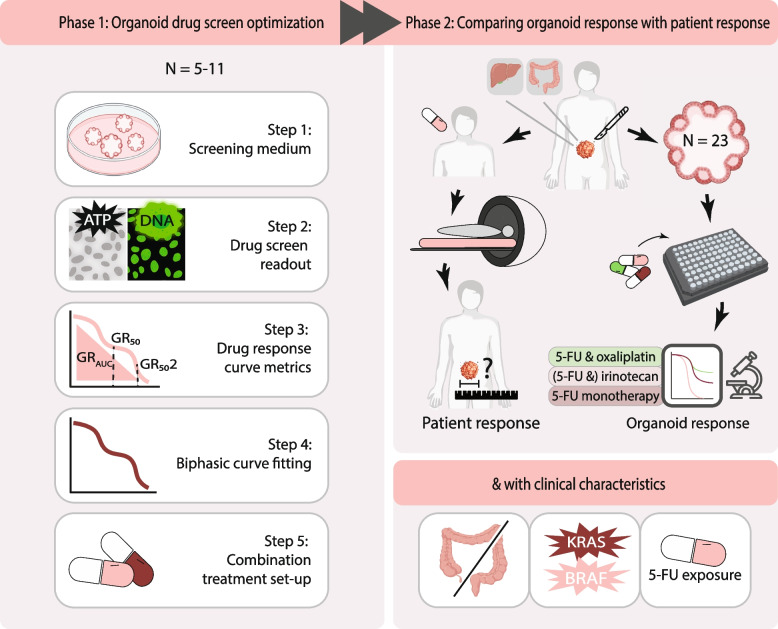
Schematic overview of the study. Tissue from metastatic and primary CRC tumours was obtained via resection or biopsy prior to starting a new line of systemic treatment. PDOs were cultured and screened for standard-of-care treatments while the patient received standard systemic treatment. PDO drug screens were optimized by comparing various methods using a limited set of 5–11 PDOs, either among one another or by correlation with patient response. For all patients, organoid and patient response were compared for treatment given after the organoid was established

We included organoids of 23 patients based on the following criteria: diagnosed with mCRC, tissue for organoid culture was obtained prior to receiving a new line of standard-of-care systemic therapy for metastatic disease (either from primary tumour or metastatic disease) and available clinical data (including treatments given, response scans and vital status). Tissue samples were collected during surgery or fine-needle biopsies within the Biobanking protocol HUB-Cancer (TCBIO #12–093, *n* = 15) or within the prospective clinical trial Organoids to Predict Therapy Response In Colorectal Cancer (OPTIC [29] #17–356, *n* = 8). Written informed consent was obtained prior to study inclusion. We first optimized drug screen methods, by examining the optimal screening medium, readout type, curve fitting, drug response curve (DRC) metrics and combination screen set-up, and we evaluated whether the optimization steps affect the correlation of the PDO screens with patient response (Fig. 1). After drug screen optimization on 5–11 PDOs per condition, we expanded the screens for the complete set of 23 PDOs and assessed the correlation with clinical response per treatment category. Additionally, we evaluated organoid sensitivity in relation to prior exposure to chemotherapy, mutational status and sidedness.

### Clinical data collection

Clinical data was collected from the electronic patient database by data managers who were blinded for the drug screen response data. The following variables were collected: sex, date of primary and metastatic disease diagnosis, number and location of metastases upon diagnosis of metastatic disease, TNM status, mismatch repair status, mutational status of the tumour (including *RAS* and *BRAF* status), and sidedness of the primary tumour (defined as right-sided (coecum-transverse colon), left-sided (splenic flexure-sigmoid) and rectosigmoid/rectal. The mutation status has been determined by NGS panels Ion Torrent PGM Cancer Hotspot panel v2Plus/Plus2/Plus3, mMIPs PATH gene panel combined with SeqNext or AvL Panel V1.2. Class I and II *BRAF* mutations were considered pathogenic, but not Class III [30]. *RAS* mutations included *KRAS* amplifications. Treatment information was collected, including primary tumour resection, local treatment of metastases (including metastasectomy or ablation), adjuvant chemotherapy (type treatment received), and the type and duration of treatment for each systemic palliative therapy line given. Chemotherapy exposure prior to organoid establishment was defined as any given systemic treatment including adjuvant treatment. A radiologist, who was blinded for the organoid screen data, reassessed all CT scans, for each treatment line according to RECIST 1.1. Clinical response on treatment was defined as the percentage size change of all target lesions during treatment, compared to the baseline scan. Progression-free survival for each treatment line was determined as the days between the start of a given treatment and the date of progressive disease on the response scan. If a patient received local treatment for metastatic target lesions, the progression-free survival date was censored for the date of the local treatment.

### Organoid culture isolation and establishment

PDO isolation was performed as van de Wetering et al. (2015) described [10]. PDOs were passaged using mechanical dissociation by pipetting and enzymatic dissociation with TrypLE Express (Gibco, Breda, the Netherlands, #12604021) for 5–10 minutes at 37 °C, washed using organoid culturing medium and re-plated by embedding in a solution of ice-cold Matrigel (Corning, Amsterdam, the Netherlands, #356231) (70%) and organoid culturing medium (Supplementary Table 1, Additional file 1) and plated as droplets on a pre-warmed 6-well plate. After solidification of the Matrigel, organoid culturing medium, containing Rho-kinase inhibitor (10 μM, Abmole Bioscience, Brussels, Belgium, #M1817), was added to the plates. The organoid culture medium was refreshed three times per week. Organoid identity was confirmed using a single-nucleotide polymorphism (SNP) array targeting 64 SNPs using TagMan OpenArray technology in combination with the QuantStudio 12 K Flex Real-Time PCR System at the Utrecht Sequencing Facility (USEQ). We calculated genetic distances of PDO DNA versus blood DNA, assigning values based on SNP comparisons (1 for XX vs XY and YY vs XY, 2 for XX vs YY, − 2 for no call, and ± 0.333 for invalid calls). PDOs with a genetic distance < 5 were included in the drug screens.

### Organoid in vitro drug screens

The timeline of the drug screen was as follows: On day − 1, PDOs were sheared and re-plated. Day 0, PDOs were harvested by incubating with 1 mg/mL Dispase II (Life Technologies Europe B.V., Zuid-Holland, the Netherlands, #17105041) for 30 minutes at 37 °C, washed twice using organoid culturing medium with Rho-kinase inhibitor, filtered using a 100 and 20 μm mesh filter to collect the PDOs which have passed through the 100 μm filter but not through the 20 μm filter, to remove debris and single cells. PDOs were resuspended in organoid screening medium with Rho-kinase inhibitor (5 μM, Abmole Bioscience, Brussels, Belgium, #M1817) with 5% Matrigel® for a final 125,000 PDOs/20 mL concentration. The organoid screening medium was composed of the organoid culture medium (Supplementary Table 1, Additional file 1) with the following adjustments: 1 ng/mL human recombinant EGF (Sigma-Aldrich, #A9165) and 10 ng/mL heregulin (Peprotech, London, UK, #100–03) to avoid interference by epidermal growth factor (EGF) with panitumumab sensitivity. Using an automated Multidrop™ Combi Reagent Dispenser, 40 μL of PDO suspension was dispensed in clear-bottomed, black-walled 384-well plates with ultra low-attachment coating (Corning, Zuid-Holland, the Netherlands, #4588). Drug concentrations were selected to obtain a complete drug response curve for the majority of PDOs, ranging from no growth inhibition at the lowest concentrations and near complete cell death at the highest concentrations. Drug concentration ranges included concentrations within the clinically relevant range [31]. Drug concentrations in combination screens were selected based on equal contribution in growth inhibition of both compounds. A 10-point concentration range of the treatments (Supplementary Table 2, Additional file 1), the positive control (Staurosporine) and negative control 1% (Dimethyl sulfoxide, Phosphate Buffered Saline or combination depending on the solvent used), were dispensed in technical quadruplicates using a Tecan Fluent liquid handler or Tecan D300E dispenser. The following commonly used CRC treatments were screened: 5-fluorouracil (5-FU), SN-38 (active metabolite of irinotecan), oxaliplatin, panitumumab, combination treatment 5-FU & oxaliplatin, and combination treatment 5-FU & SN-38. On day 5, readouts were obtained by quantifying cell viability using CellTiter-Glo 3D (Promega, #G9681, 40 μL/well) with a Tecan Spark plate reader and CyQUANT Direct proliferation assay (Invitrogen, C7026, 20 μL/well) with Perkin Elmer *Operetta*® CLS™. A baseline readout was measured on day 0 in a separate plate with PDOs without treatment. Drug screens were performed in at least two duplicate experiments on different days. Percentage viability and growth rate inhibition (GR) metrics values were calculated [32]. GR values range from 1 to − 1, with 0 to 1 for partial growth inhibition, 0 for complete cytostasis, and 0 to − 1 for cell death. Four parameter log-logistic curves and biphasic sigmoid curves were fit using the DRC package in R [33] and the biphasic dose-response model in GraphPad, respectively. We calculated the Akaike information criterion (AIC) values of both models for 5-FU & oxaliplatin, to objectify goodness-of-fit. As AIC values of the biphasic model were lower (median decrease 9.5, range 2.5–26.7), response curves for 5-FU & oxaliplatin were fit using a biphasic model instead of a log-logistic model, resulting in GR_50_1 and GR_50_2 parameters. The GR_AUC_ (area under the non-fitted ‘curve’ of the raw GR values), GR_50_ (concentration that gives half-maximal growth rate inhibition), GR_50_1 and GR_50_2 (concentration of 50% growth rate inhibition of the upper and lower part of the biphasic curve, respectively) were calculated. Normalized values for GR_AUC_ and GR_50_(2) were calculated using the maximum and minimum measured parameter per treatment to compare different treatment types with different concentration ranges.

### Statistical methods

Drug screens were optimized by comparing correlation coefficients of different PDO screening methods with clinical response. Where applicable, two-sided Pearson correlation tests were utilized to examine linear associations between continuous variables. Spearman correlation tests were employed when the data did not meet the assumptions of normality. Associations between clinical response and organoid response were examined by scatterplots. Univariate cox proportional hazard regression analysis was performed to estimate the hazard ratio (HR) with a 95% CI. AUC values were transformed to a standard normal distribution to estimate the HR. Kaplan-Meier survival curves were used to plot progression-free survival, and a two-sided Wald test was used to compare survival between patients with different organoid sensitivity. PDOs were classified as sensitive if the normalized GR_AUC_ of the 5-FU & oxaliplatin drug screen was below the upper tertile. This cut-off was based on the response rate of approximately 60% for patients treated with FOLFOX in the first line in order to evaluate whether the upper third most resistant PDOs indeed predict failure of FOLFOX [1, 2]. We evaluated organoid sensitivity in relation to prior exposure to chemotherapy, mutational status and sidedness using boxplots. A two-sided Mann-Whitney U-test were applied for comparing 2 groups. Analysis was performed in R (version 4.0.3). DRCs and derived metrics were fit and calculated with the GRmetrics (version 1.16.0) and DRC (version 3.0–1) packages. Heatmaps were plotted using the Pheatmap (version 1.0.12) package.

## Results

### Patient cohort

The study cohort consists of 23 mCRC patients who were treated with standard-of-care systemic therapy for metastatic disease. Four PDOs were derived from primary tumours and 19 from liver metastases (Table 1). Most patients were chemotherapy-naïve when the PDO was established (14/23). The clinical characteristics of the included patients reflect the heterogeneous mCRC population (Supplementary Table 3, Additional file 1). The cohort includes 10 *RAS*-mutants, three *BRAF*-mutants and 10 *RAS/BRAF*-wildtype tumours. The cohort comprises five patients with rectal tumours, 11 patients with left-sided tumours, and seven patients with right-sided tumours (Supplementary Table 3, Additional file 1). Evaluable treatments in the first line of treatment after PDO establishment include capecitabine monotherapy (prodrug of 5-FU, *n =* 6), 5-FU & oxaliplatin combination treatment (*n =* 13) and irinotecan-based treatment (5-FU & irinotecan combination treatment or irinotecan monotherapy, *n =* 4). Late-line treatments include 5-FU & oxaliplatin combination treatment (*n* = 1), irinotecan-based treatment (*n* = 6) and panitumumab (*n* = 6). Bevacizumab treatment was given to the vast majority of the patients (*n* = 19). However, this treatment is not evaluable in vitro, since its main effect is on the tumour vasculature [34].

**Table 1:** Overview of evaluable treatments per patient

Patient ID	Evaluable treatment 1	Response 1	Evaluable treatment 2	Response 2	Evaluable treatment 3	Response 3	Mutational status	PDO origin	Stage at presentation	Sidedness
04	5-FU	SD	5-FU & irinotecan	SD	5-FU & oxaliplatin	PR	RAS mutant	Metastatic	IV	Left
06	5-FU	SD					RAS mutant	Metastatic	IV	Left
07	5-FU	SD					RAS mutant	Metastatic	IV	Left
11	5-FU	PD					Wildtype	Metastatic	IV	Right
16	5-FU	SD	Panitumumab	PD			BRAF mutant	Primary	IV	Right
18	5-FU	PD					Wildtype	Metastatic	III	Rectum
01	Irinotecan	SD					RAS mutant	Metastatic	II	Rectum
02	5-FU & irinotecan	SD					RAS mutant	Metastatic	III	Right
23	5-FU & irinotecan	SD	Panitumumab	SD			BRAF mutant	Metastatic	III	Left
20	5-FU & irinotecan	PD								
20	5-FU & oxaliplatin	PD					RAS mutant	Primary	IV	Left
03	5-FU & oxaliplatin	PD					RAS mutant	Primary	IV	Right
05	5-FU & oxaliplatin	PR					RAS mutant	Primary	IV	Right
08	5-FU & oxaliplatin	PR			Panitumumab	SD	Wildtype	Metastatic	IV	Rectum
09	5-FU & oxaliplatin	SD					BRAF mutant	Metastatic	IV	Left
10	5-FU & oxaliplatin	PR	Panitumumab	SD			Wildtype	Metastatic	IV	Left
12	5-FU & oxaliplatin	PR	5-FU & irinotecan	SD	Panitumumab	SD	Wildtype	Metastatic	IV	Left
13	5-FU & oxaliplatin	SD	Irinotecan	SD			RAS mutant	Metastatic	IV	Left
14	5-FU & oxaliplatin	PR	5-FU & irinotecan	PR			Wildtype	Metastatic	III	Rectum
15	5-FU & oxaliplatin	PR	5-FU & irinotecan	SD	Panitumumab	SD	Wildtype	Metastatic	III	Rectum
17	5-FU & oxaliplatin	PR	5-FU & irinotecan	SD			RAS mutant	Metastatic	IV	Right
19	5-FU & oxaliplatin	PD					Wildtype	Metastatic	III	Left
21	5-FU & oxaliplatin	PD					Wildtype	Metastatic	II	Right
22	Panitumumab	PR					Wildtype	Metastatic	IV	Left

Table describing the evaluable treatments, ranked per primary evaluable treatment. The first, second and third lines after organoid establishment are shown for each patient, along with the patient’s best RECIST response during treatment. The *RAS/BRAF* mutational status of the original tumour, PDO origin, tumour stage at presentation and primary tumour location (sidedness) are presented. All patients had a proficient mismatch repair (pMMR) tumour. Patient 20 was resistant to FOLFOXIRI triplet chemotherapy and is, therefore, evaluable for 5-FU & oxaliplatin and 5-FU & irinotecan. Clinically capecitabine (prodrug 5-FU) is used often, while in in vitro screens 5-FU is evaluated

*5-FU* 5 Fluorouracil, *CAP* Capecitabine, *ID* Identification, *PD* Progressive disease, *PDO* Patient-derived organoid, *PR* Partial response, *SD* Stable disease

### Organoid drug screen quality

The drug screen quality was analyzed by examining the Z’-factor [35], correlation in DRC metrics among the biological replicates and Bland-Altman plot for the difference in GR_AUC_ between replicates. The mean Z’-factor for all PDOs and treatments was 0.64 with only 4% below 0.5, indicating a good quality drug screen (Supplementary Table 4, Additional file 1). Biological replicates were not included in the analysis if the runs had a Z’-factor < 0.3 or if technical errors had occurred (e.g. dispensing error). When drug suspension errors occurred or replicate curves deviated, the respective biological replicates were excluded (9% of the drug screens) and a third and/or fourth run was performed. The calculated DRC metrics GR_AUC_ GR_50_ and GR_max_ of different included biological replicates were significantly correlated (Spearman correlation > 0.84, *p* < 0.05, (Supplementary Fig. 1A, Additional file 1). The mean difference in GR_AUC_ between included replicates was − 0.09, with limits of agreement ranging from − 1.17 to 0.98 (Supplementary Fig. 1B, Additional file 1). The DRC per PDO for each treatment screened are displayed in Supplementary Fig. 2A, B, Additional file 1.

### Phase 1: drug screen optimization

#### Step 1: removal of N-acetyl cysteine from screening medium

We compared screening medium with and without NAC for oxaliplatin-based growth inhibition assays. We confirmed that NAC increased resistance to oxaliplatin-based treatment, with a 24% increase in the median GR_AUC_ (Supplementary Fig. 3A, Additional file 1). Withdrawal of NAC from the PDO drug screening medium had no disadvantageous effect on organoid growth of the untreated control, with a growth rate from day 0 to day 5 of 5.5 with NAC (95% CI 4.2, 6.7) and 6.8 without NAC (95% CI 5.5, 8.1). Moreover, the correlation with clinical response improved after removal of NAC (Pearson correlation 0.77 without NAC vs 0.3 with NAC, Table 2). We, therefore, continued all drug screens in the following steps for oxaliplatin-based treatment without NAC.

**Table 2:** Comparison of organoid drug screening conditions

Comparison	Condition 1	Condition 2
Screening medium 5-FU & oxaliplatin	With NAC (CQ ratio, GR) r = 0.32 [− 0.78, 0.94]	Without NAC (CQ ratio, GR) r = 0.77 [− 0.35, 0.98]
Readout 5-FU & oxaliplatin	CQ (ratio without NAC, GR) r = 0.77 [− 0.35, 0.98]	CTG (ratio without NAC, GR) r = 0.81 [− 0.24, 0.99]
Drug response curve metrics: Viability vs GR 5-FU & oxaliplatin	Viability (ratio without NAC) r = 0.46 [− 0.19, 0.83]	GR (ratio without NAC) r = 0.6 [− 0.01, 0.88]
Drug response curve metrics: Viability vs GR 5-FU & SN-38	Viability (CTG fixed) r = 0.26 [− 0.54, 0.82]	GR (CTG fixed) r = 0.66 [− 0.08, 0.93]
Drug response curve metrics: Viability vs GR 5-FU	Viability (CTG) r = 0.3 [− 0.68, 0.89]	GR (CTG) r = 0.58 [− 0.44, 0.95]
Combination treatment set-up 5-FU & oxaliplatin	Fixed (CTG with NAC, GR) r = − 0.25 [− 0.76, 0.45]	Ratio (CQ without NAC, GR) r = 0.6 [− 0.01, 0.88]
Combination treatment set-up 5-FU & SN-38	Fixed (CTG, GR) r = 0.66 [− 0.08, 0.93]	Ratio (CTG, GR) r = 0.3 [− 0.79, 0.94]

Pearson correlation coefficients of PDO response with patient response for different PDO drug screening conditions, with 95% confidence interval

*5-FU* 5 Fluorouracil, *CTG* CellTiter-Glo, *CQ* CyQUANT, *GR* Growth rate, *NAC* N-acetyl cysteine, *SN-38* active metabolite of irinotecan

#### Step 2: drug screen readout (CellTiter-Glo versus CyQUANT)

Various approaches exist for quantifying organoid viability as a proxy for drug response. We used ATP-based CellTiter-Glo, as this luminescence-based readout is most commonly used in literature as it is a rapid, high-throughput readout [36]. In addition, we explored the nuclear fluorescent-based CyQUANT readout. CyQUANT offers the benefit of directly measuring number of viable cells instead of relying on surrogate measures [37]. We found that the Pearson correlation between drug sensitivity measurements (GR_AUC_) utilizing CellTiter-Glo and measurements utilizing CyQUANT was 0.78 for 5-FU monotherapy (95% CI 0.50, 0.92)*,* 0.69 for 5-FU & SN-38 combination treatment (95% CI -0.04, 0.94) and 0.57 for 5-FU & oxaliplatin combination treatment (95% CI -0.23, 0.91 Supplementary Fig. 3B-D, Additional file 1). As drug sensitivity measurement for 5-FU & oxaliplatin combination obtained from the CyQUANT readout showed limited concordance with the CellTiter-Glo readout, we further explored the CyQUANT readout for reflecting patient response to this drug combination in 6 PDOs. We found that CyQUANT and CellTiter-Glo measurements showed similar Pearson correlations with patient response of 0.77 and 0.81, respectively (Table 2).

#### Step 3 and 4: viability, growth rate metrics and curve fitting

**Fig. 2:**
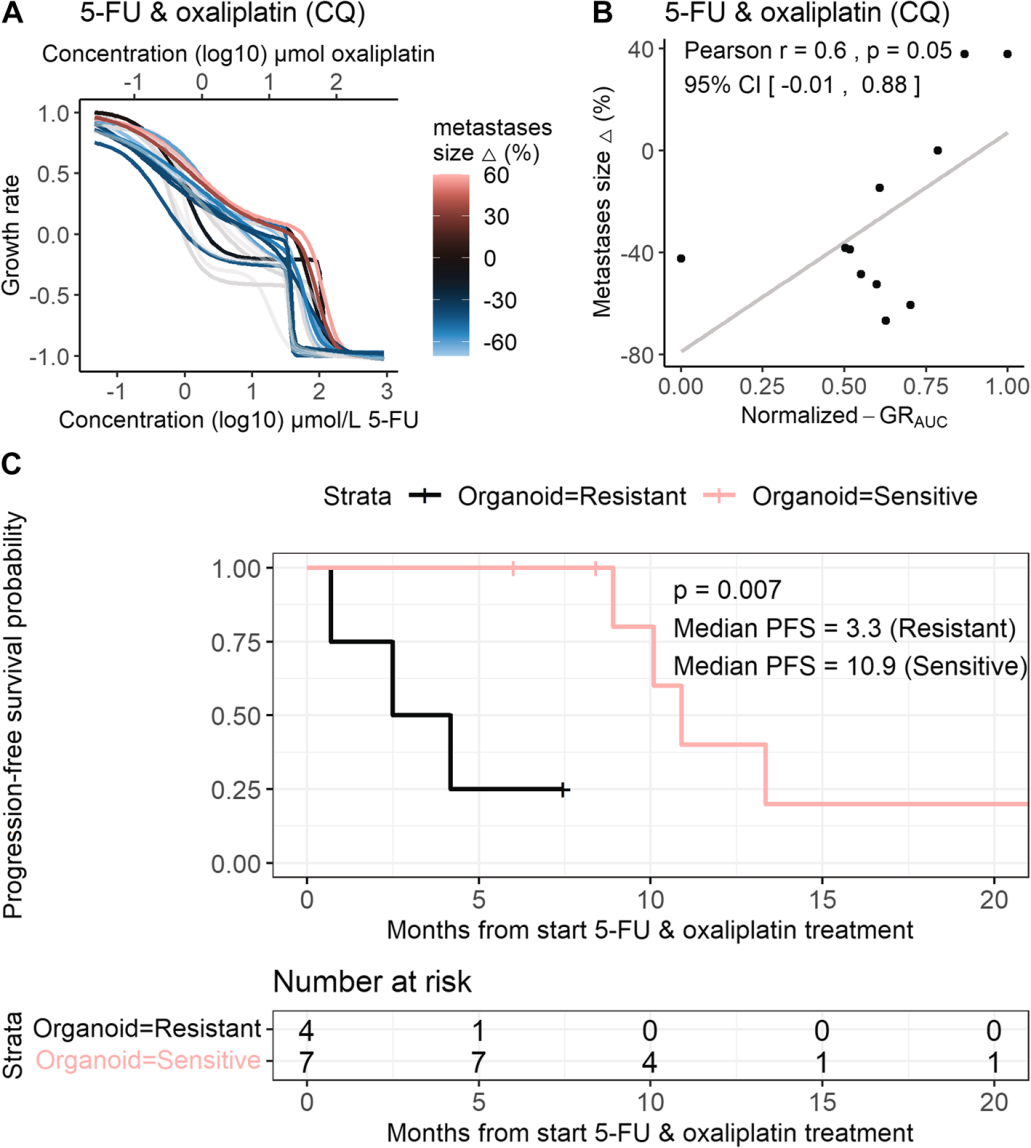
Association of tumour size change and PFS with organoid response to 5-FU & oxaliplatin. **A** DRCs of organoid sensitivity to 5-FU & oxaliplatin combination treatment screened in a 1.8:1 ratio. **B** Scatterplots show the correlation between patient response (% size change during treatment) and organoid response measured (GR_AUC_) for 5-FU & oxaliplatin combination treatment screened in a 1.8:1 ratio. **C** Kaplan-Meier progression-free survival curves of patients stratified by organoid sensitivity to 5-FU & oxaliplatin, based on normalized GR_AUC_ (cut-off upper tertile = 0.63)_._ Censoring events are indicated by vertical bars on the corresponding curve. The table underneath each plot denotes the numbers at risk. Log-rank test-based *p* value is shown. *Abbreviations:* 5-FU (5-fluorouracil), DRC (drug response curve), GR_AUC_ (area under the growth rate inhibition curve), PFS (progression-free survival)

For all evaluated treatments, we compared GR metrics to percentage viability measurements (Supplementary Fig. 3E, Additional file 1). Using GR metrics instead of percentage viability corrects for confounders in organoid drug sensitivity, related to differences in cell division rate in untreated controls. Applying GR metrics improved correlations with patient response compared to percentage viability for 5-FU & oxaliplatin (Pearson correlation 0.6 vs 0.46), 5-FU & SN-38 combination treatment (Pearson correlation 0.66 vs 0.2) and 5-FU monotherapy (Pearson correlation 0.58 vs 0.30, Table 2). Response curves of most drugs exhibit a sigmoidal shape. Interestingly, we observed a biphasic drug response with a static plateau for 5-FU & oxaliplatin combination screens (Fig. 2A).

#### Step 5: combination treatment set-up, ‘fixed’ versus ‘ratio’

Finally, we evaluate the most optimal strategy for combination screens for respectively 5-FU & oxaliplatin and 5-FU & SN-38 as these drugs are standardly used in combination in clinical practice. We compared combination drug screens using a (fixed) ratio with increasing dosage of both 5-FU & oxaliplatin (‘ratio’) versus using a fixed concentration of oxaliplatin and increasing only 5-FU (‘fixed’). Applying drugs in a ‘ratio’ versus ‘fixed’ combination influenced the correlation with clinical response and results were contradictory for oxaliplatin and irinotecan combination treatment. For oxaliplatin, using a fixed concentration of 0.05 μM oxaliplatin and increasing 5-FU (‘fixed’) did not show a positive correlation with patient response to 5-FU & oxaliplatin, as indicated by a Pearson correlation of − 0.25, versus 0.6 when screened in a 1.8:1 ratio, Table 2. For irinotecan, using a fixed concentration of 0.01 μM SN-38 and increasing 5-FU (‘fixed’) resulted in a Pearson correlation of 0.66, versus a 0.3 when screened in a 1500:1 ratio (Table 2).

### Phase 2: correlation of organoid response using the optimized drug screen methods with patient response

Next, the optimized screening methods were used to examine the association between organoid and patient response on the complete set of PDOs per treatment type, for 5-FU & oxaliplatin, irinotecan-based treatment and 5-FU monotherapy.

## 5-FU & oxaliplatin

We expanded the CyQUANT screens for evaluating organoid sensitivity and correlation with patient response to 5-FU & oxaliplatin on 14 PDOs, of which 11 PDOs were derived from metastatic lesions. 5-FU & oxaliplatin comprises the largest treatment cohort and the majority of patients was treated in the first line (*n* = 13), directly after the organoid biopsy was obtained. Sensitivity of PDOs derived from metastatic lesions to 5-FU & oxaliplatin without NAC, screened in a 1.8:1 ratio and measured by GR_AUC_, showed a Pearson correlation of 0.6 (*p* = 0.053, Fig. 2A, B) with change in tumour size during 5-FU & oxaliplatin treatment. The median GR_AUC_ was 0.57 for PDOs derived from patients with a decrease in size of metastatic lesions and 0.87 for PDOs derived from patients with stable or an increase in size of metastatic lesions (*p =* 0.012). There was discordance between patient response to 5-FU & oxaliplatin and PDO response in two of the three PDOs derived from primary tumours. The Pearson correlation with patient response for PDOs derived from metastases and primary tumours was limited to 0.29 (Supplementary Fig. 4A, Additional file 1). Next to GR_AUC_, other DRC metrics were evaluated, including the GR_50_2 parameter for biphasic curves. GR_50_2 showed a Pearson correlation of 0.7 (*p* = 0.02) with patient response to 5-FU & oxaliplatin (Supplementary Fig. 4B, Additional file 1). We explored PFS as a secondary outcome measure for patient response. First, in a Cox proportional hazards model, the hazard ratio was estimated to be 2.30 (95% CI 0.68, 7.71, *p* = 0.2). Secondly, PDOs were classified as sensitive or resistant based on the GR_AUC_. PFS of patients with PDOs classified as sensitive was substantially longer compared to patients with resistant PDOs (median PFS 3.3 vs 10.9 months, log rank *p* = 0.007, Fig. 2C).

## Irinotecan-based treatment

**Fig. 3:**
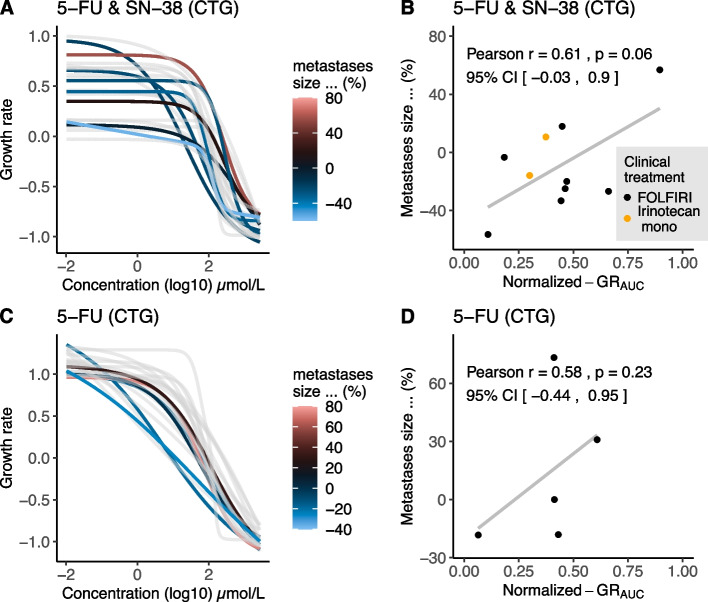
Association of tumour size change and organoid response to irinotecan-based treatment and 5-FU monotherapy. **A** DRCs of organoid sensitivity to 5-FU & SN-38 combination treatment. **B** Scatterplots show the correlation between patient response (% size change during treatment) and organoid response measured (GR_AUC_) for (5-FU &) SN-38. **C** DRCs of organoid sensitivity to 5-FU monotherapy. **D** Scatterplots show the correlation between patient response (% size change during treatment) and organoid response measured (GR_AUC_) for 5-FU monotherapy. *Abbreviations:* 5-FU (5-fluorouracil), DRC (drug response curve), CTG (CellTiter-Glo), GR_AUC_ (area under the growth rate inhibition curve), SN-38 (active metabolite of irinotecan)

Ten patients received irinotecan-based treatment in 1st or 2nd treatment line, either monotherapy (*n* = 2) or in combination with 5-FU (*n* = 8). For irinotecan-based treatment, organoid sensitivity to a fixed dose SN-38 and increasing concentrations 5-FU (with NAC, using CellTiter-Glo) measured by GR_AUC_ showed a Pearson correlation of 0.61 (*p* = 0.059) with patient response (Fig. 3A, B). For 5-FU & SN-38 no other DRC parameters then GR_AUC_ were suitable for measuring drug sensitivity. GR_50_ could not reliably be measured due the absence of an evident lower plateau in the DRC of these screens (Fig. 3A). As patients in this cohort were treated in different lines, PFS could not reliably be compared.

## 5-FU

Six patients were treated with capecitabine monotherapy (prodrug of 5-FU) which allows a direct single agent comparison of patient response with organoid response to 5-FU. All six patients received capecitabine as 1st treatment line and directly after the organoid biopsy was obtained. Organoid sensitivity to 5-FU (with NAC, using CellTiter-Glo) measured by GR_AUC_ showed a Pearson correlation of 0.58 (*p* = 0.23) with % change in tumour size during capecitabine treatment (Fig. 3C, D).

### PDOs reflect clinical heterogeneity in drug sensitivity, effect of prior treatment, primary tumour sidedness and mutational status

**Fig. 4:**
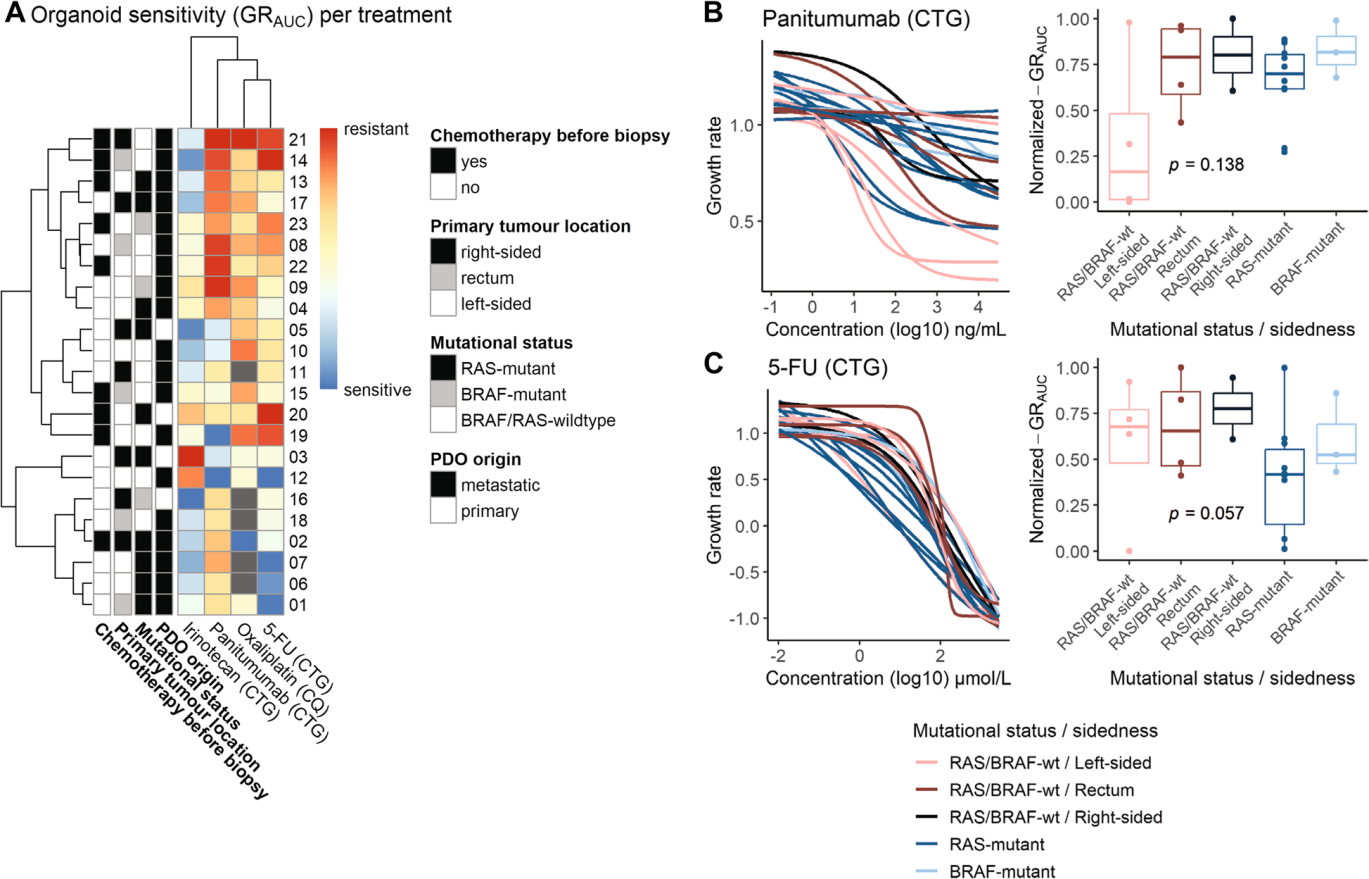
Organoid sensitivity based on primary tumour location and mutational status. **A** Clustered heatmap of the normalized GR_AUC_, with characteristics of the patient indicated in the first four columns for chemotherapy exposure prior to PDO establishment (adjuvant or first line), the primary tumour location (left- versus right-sided), mutational status (*BRAF-*mutant, *RAS-*mutant and *RAS/BRAF-*wildtype) and PDO origin (metastatic and primary tumour). The normalized GR_AUC_ are illustrated as a heatmap with a column for each treatment type examined, with PDOs that were not screened for oxaliplatin in grey. **B** and **C**) The DRCs for panitumumab (**B**) and 5-FU (**C**) treatment and boxplots of the GR_AUC_ for PDOs categorized according to the tumour’s mutational status and sidedness (*RAS/BRAF*-wildtype and left-sided; *RAS/BRAF-*wildtype and rectal; *RAS/BRAF*-wildtype and right-sided; *RAS*-mutant and *BRAF*-mutant. Boxplots show the minimum, median, maximum, upper and lower quartiles and individual data points. *Abbreviations:* 5-FU (5-flouruoracil), DRC (drug response curve), CTG (CellTiter-Glo), CQ (CyQUANT), GR_AUC_ (area under the growth rate inhibition curve), PDO (patient-derived organoid), SN-38 (active metabolite of irinotecan), wt (wildtype)

Finally, as all PDOs in the cohort were screened for commonly used treatments in CRC, allowing comparison of drug sensitivity for patients with different tumour characteristics. We investigated the impact of mutational status, primary tumour location, and prior chemotherapy exposure on organoid drug sensitivity. When we examined organoid sensitivity for different standard-of-care drugs, a wide range of responses was seen (Fig. 4A). Some PDOs display a generally resistant phenotype (e.g. PDO 21) to most treatments, while other PDOs display a differential treatment response (e.g. PDO 19). We found that PDOs obtained from patients with a left-sided *RAS/BRAF*-wildtype tumour (*n* = 4) were more sensitive to EGFR-inhibitor panitumumab, compared to PDOs obtained from patients with right-sided or rectal *RAS/BRAF*-wildtype tumours, *RAS*-mutant tumours and *BRAF*-mutant tumours (*n* = 19, median normalized GR_AUC_ 0.17 vs 0.74, *p* = 0.138). Interestingly, we observed varying organoid sensitivity to panitumumab within the group of left-sided *RAS/BRAF*-wildtype tumours (Fig. 4B). Organoid sensitivity to chemotherapeutic treatment with 5-FU was increased in organoids derived from *RAS* mutant tumours (*n* = 10, median normalized GR_AUC_ 0.42 vs 0.64, *p* = 0.057, Fig. 4C).

**Fig. 5:**
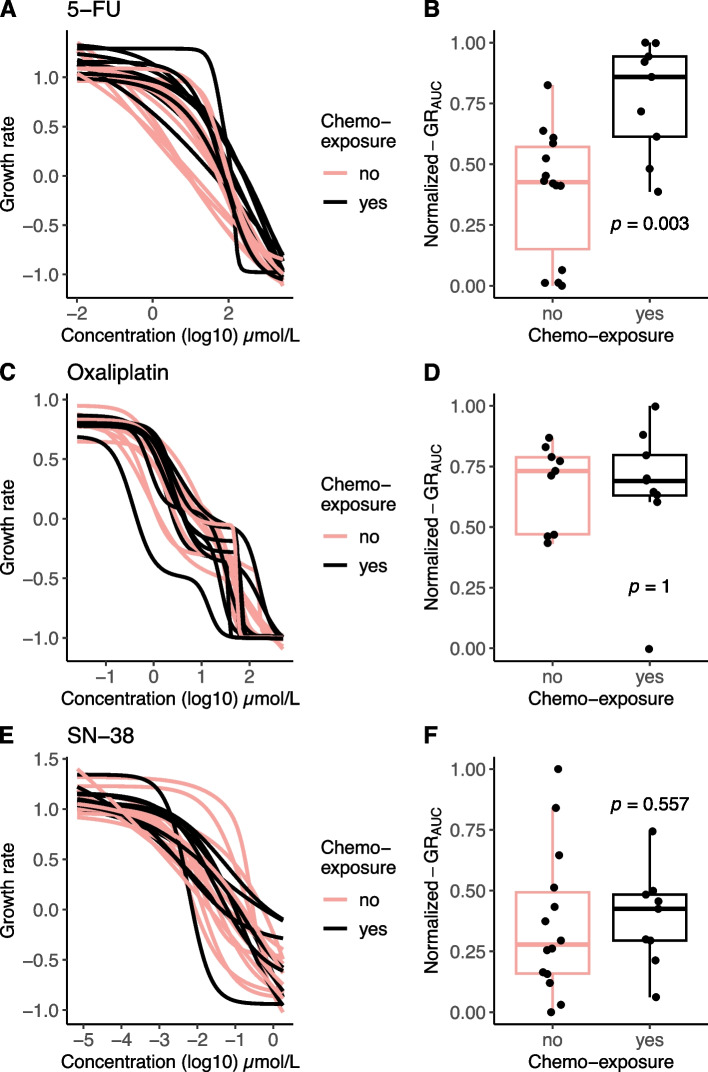
Increased resistance to 5-FU after prior exposure to chemotherapy. The association between prior chemotherapy exposure to patients before the biopsy for PDOs was established and organoid response (GR_AUC_) is shown for 5-FU, oxaliplatin and SN-38. **A**, **C** and **D**. The DRC of organoid sensitivity with red curves indicating 5-FU or capecitabine containing chemotherapy exposure. **B**, **D** and **F** Boxplots of normalized GR_AUC_ for patients that were exposed to chemotherapy versus chemotherapy-naïve patients. Boxplots show the minimum, median, maximum, upper and lower quartiles and individual data points. *Abbreviations:* 5-FU (5-flouruoracil), GR_AUC_ (area under the growth rate inhibition curve), SN-38 (active metabolite of irinotecan)

PDOs from tumours exposed to 5-FU or capecitabine (in combination with oxaliplatin) before establishment (*n* = 9), mainly in the form of adjuvant treatment (*n* = 6), are more resistant to 5-FU than untreated PDOs (median normalized GR_AUC_ of 0.86 versus 0.43, *p* = 0.003). However, this resistance does not extend to other chemotherapy types (Fig. 5).

## Discussion

Our study shows that CRC PDO drug screens need optimization and standardization before being reliably utilized as biomarker for patient response in clinical practice. A standardized screening protocol could enhance cross-study comparisons and improve reproducibility as significant variation exists regarding screening methods used in different studies [7, 24]. To establish a drug screening method that adequately reflects the clinical situation, we used an inverse approach. Starting from the patient’s response, we fine-tuned different parameters of the drug sensitivity assays in a small population. This enhanced the accuracy PDOs in reflecting the patient’s sensitivity. For subsequent evaluation of the predictive value of the optimized screening method in the complete set, we used a cohort of heterogeneous PDOs, well reflecting the clinical population. We optimized the drug screening method through five steps, for which the results are summarised in Table 3. The first step regards the medium composition, as it is already known that changing medium components can have dramatic effects on chemosensitivity [38]. It was previously reported that specifically NAC affects sensitivity to oxaliplatin in CRC organoids [28]. This can be explained by the fact that NAC causes detoxification of oxaliplatin through glutathione synthesis and can, therefore, interfere with organoid sensitivity to platinum-based chemotherapy, yet does not affect sensitivity to 5-FU or irinotecan [27]. In line with these results, our screens confirmed increased resistance to oxaliplatin-based treatment with NAC-containing screening medium. This led to the necessity of using high, clinically irrelevant oxaliplatin doses and we found a reduced correlation with clinical response when NAC was added to the screening medium [31]. Notably, studies that use NAC in oxaliplatin screening found no correlation between organoid sensitivity and patient response [20, 23]. Therefore, we recommend removing NAC from the medium in oxaliplatin-based drug screens. Secondly, we compared two types of drug screen readouts to define the most optimal readout. The ATP-based readout is most commonly used for PDO drug screening. Nevertheless, based on the compound mechanism of action, ATP measurements can lead to misleading results as metabolic activity does not always correlate with cell viability [36]. Therefore, we explored the use of a well-established readout based on DNA-content for 2D cell lines (CyQUANT) to measure cell viability in PDO-based screens. Nuclear fluorescent-based assays such as CyQUANT or propidium iodide dye combined with Hoechst are less affected by cell changes unrelated to viability, such as senescence. Propidium iodide and Hoechst have previously demonstrated their suitability for high-throughput screening of colorectal adenoma PDOs [39]. CyQUANT was used previously in a prospective organoid-guided interventional trial with positive results, albeit with modest clinical benefit [40]. We confirmed that drug sensitivity measurements with the CyQUANT readout instead of the ATP-based CellTiter-Glo readout was possible in 3D-based screens and did not affect the correlation with patient response. CyQUANT may, therefore, serve as promising alternative drug screening method, enabling screening with fewer than 10 organoids per well. Thirdly, in line with previous research showing that bias caused by the proliferation rate of PDOs can be avoided by using GR metrics for organoid response analysis [32], we confirm that using GR metrics improved the correlation with patient response. Despite good correlations of PDO and clinical response in studies employing both percentage viability and GR metrics [6, 7], we found that GR metrics showed better correlation with patient response than percentage viability metrics. Previous studies used DRC parameters AUC, IC_50_, GR_50_ or GR_max_ to evaluate organoid response [14–16, 23]. In our research, GR_AUC_ proved robust and patient-response reflective. For full sigmoidal/biphasic curves, GR_50_(2) could also serve as reliable drug sensitivity measures. Fourthly, the conventional approach in all previous studies involved log-logistic curve fitting. Nonetheless, goodness-of-fit improved by applying biphasic curve fitting for 5-FU & oxaliplatin in our study. Finally, we optimized the drug screen set-up for combination screens and found that a fixed concentration of SN-38 is recommended for SN-38 based combination screening. This might be explained by the fact that 5-FU can inhibit the in vitro efficacy of SN-38 at high concentrations [41], although one study showed positive results where 5-FU & SN-38 was used in a ratio [21]. For oxaliplatin-based treatments, a ratio screen is preferred to capture the additive effect of both compounds. This is in line with studies that used a comparable ratio [14, 16, 22] or a drug matrix and found a good association with patient response, while no association was found in studies that used different methods for combination screens [20, 23].

**Table 3:** Recommendations for CRC organoid drug screening

Topic	Evidence	Recommendations
1. Medium composition	Resistance to oxaliplatin-based treatment increases with NAC in screening medium.	Remove NAC from screening medium in oxaliplatin-based drug screens.
2. Readouts	CellTiter-Glo measurements are in agreement with CyQUANT measurements.	Both readouts can be used. CyQUANT provides the advantage of performing drug screens with 5–10 PDOs per well.
3. DRC metrics	GR metrics showed better correlation with patient response than percentage viability metrics. GR_AUC_ is the most robust DRC metric and best reflects PDO and patient response.	Apply GR metrics to correct for confounders in organoid drug sensitivity, related to differences in natural cell division rate. Employ GR_AUC_ for comparison with patient response, or GR_50_(2) if a clear lower curve plateau is present.
4. DRC fitting	PDOs exhibit a biphasic drug response to 5-FU & oxaliplatin.	Apply a biphasic model for DRC fitting instead of a log-logistic model.
5. Combination treatment set-up	SN-38 & 5-FU in a ratio combination screen did not reflect patient response to 5-FU & irinotecan. Oxaliplatin & 5-FU with a fixed oxaliplatin concentration did not reflect patient response to 5-FU & oxaliplatin.	Use a fixed concentration of SN-38 and increase the 5-FU concentration for 5-FU & SN-38 combination screens. Use a concentration ratio 5-FU:oxaliplatin for oxaliplatin-based combination screens.

*5-FU* 5 Fluorouracil, *CTG* CellTiter-Glo, *CQ* CyQUANT, *DRC* Drug response curve, *GR*_*AUC*_ area under the growth rate inhibition curve, *NAC* N-acetylcysteine, *PDO* Patient-derived organoid, *SN-38* active metabolite of irinotecan

Regarding the correlation of PDO response with clinical response, our findings are in line with previous results. In a recent systematic review the overall positive predictive value for organoid informed treatment for CRC is 68% and the negative predictive value is 78% for standard of care chemotherapy, targeted therapy and radiotherapy [6]. In literature, consistent results are seen for 5-FU and irinotecan-based treatment, showing good correlations of the AUC with both RECIST response and PFS in several, relatively small, studies which align with our results [6, 7, 14–16, 23]. In our study, organoid response (GR_AUC_) positively correlated with patient response (% size change in metastatic lesions) for 5-FU & oxaliplatin, despite the small sample size. Furthermore, classifying PDOs as sensitive and resistant based on GR_AUC_, resulted in a clinically relevant difference in median PFS in the sensitive and resistant group, which is in line with previous reports including PDOs treated with 5-FU & oxaliplatin [14, 22]. In addition to the correlation with patient response, we show that CRC PDOs adequately reflect key clinical aspects regarding drug sensitivity and capture heterogeneity in treatment response. Patient response to EGFR inhibitors is influenced by factors such as tumour sidedness and *RAS/BRAF* mutational status. In line with the findings of large clinical trials [42, 43] and PDO screens [44], PDOs from patients with a left-sided colon *RAS/BRAF*-wildtype tumour were most sensitive to panitumumab in our cohort. In addition to mutational status, we showed that the resistance after prior 5-FU or capecitabine containing treatment is well captured in PDOs. This implies that PDOs provide a representative model for the 5-FU resistant state after prior chemotherapy exposure, supported by increased PDO resistance after 5-FU treatment in a recent study [45]. As recognized in daily clinical practice, adjuvant chemotherapy might also affect response to palliative treatment. In this context, PDOs could be applied to study resistance mechanisms and evasion strategies. It also underlines the importance of deriving organoids directly before a new treatment starts, as prior treatments affect sensitivity to subsequent therapies.

It is essential to acknowledge the limitations that stress the need for subsequent larger prospective studies to validate our results. In this small retrospective study utilizing biobank samples, most samples were not initially collected for direct comparison with patient response. Consequently, these samples were not acquired immediately before initiating the evaluated treatment, which compromises the accuracy of comparison with patient response. The cohort’s diversity further stems from the fact that the collected tissue does not exclusively mirror the metastatic lesions under evaluation in patient responses; it might encompass primary tissue or other resected metastases. Both may lead to an underestimation of the correlation with response. The limited number of PDOs in the treatment subgroups constrains the demonstration of significant correlations with patient response for all treatments and prohibits drawing strong conclusions. Moreover, the small sample size can lead to sampling bias with individual variations having a disproportionate impact on outcomes, and difficulty in accounting for confounding variables. The optimization of drug screen methods requires evaluation of generalizability and validation of our PFS cut-off in an independent, larger cohort before applying in other laboratories. Of note, relative PDO sensitivity is influenced by the sensitivity of other PDOs in the cohort it is compared to. To prevent misinterpretation, it is essential to compare individual drug screens to large cohorts, ensuring consistency and avoiding errors due to differences in sensitivity across groups.

Prospective validation is currently ongoing in the OPTIC trial [29]. Here, we establish PDOs for mCRC patients from newly obtained biopsies immediately prior to the start of treatment, and directly compare patient response with organoid response. Further drug screen optimization regarding duration of drug exposure and assay miniaturization [45], is key to advance towards the clinical application of PDOs for personalized treatment. PDO drug screen optimization should be extended beyond mCRC to other stages of disease and other types of cancer [46–48]. PDOs have been used for screening targeted treatments, treatment in the non-metastatic stage, such as radiotherapy for rectal cancer, and experimental treatments [49, 50]. A prior prospective study to guide experimental targeted treatments did not show clinical benefit [51], underscoring the additional need for refinement of screens for other treatment types.

## Conclusions

Our study emphasizes the critical impact of the screening methods for determining correlation between PDO drug screens and mCRC patient outcomes. We used a 5-step optimization strategy including NAC removal from the screening medium, using biphasic or logistic curve fitting based on the type of treatment, applying growth rate metrics and selecting ratio or fixed concentrations depending on the combination treatments used. By using the optimized methods, PDO response correlated with patient response for oxaliplatin-based treatment. This optimization provides a basis for future research on the clinical utility of PDO screens. Furthermore, PDOs adequately reflect the effect of prior treatment, primary tumour sidedness and mutational status, supporting their use for modeling colorectal cancer in vitro.

### Supplementary Information

**Additional file 1: Supplementary Table 1.** Composition of organoid culture medium. **Supplementary Table 2.** Chemotherapies and targeted treatments used in drug screens. **Supplementary Table 3.** Baseline characteristics of the cohort of patients. **Supplementary Table 4.** Quality control analysis of the drug screens showing the Z’-factor. **Supplementary Fig. 1.** Quality control analysis of the drug screens illustrating the difference between duplicate assays. **Supplementary Fig. 2.** Individual drug response curves for each PDO per treatment. **Supplementary Fig. 3.** Comparing different drug screening methods. **Supplementary Fig. 4.** The impact of different drug screening methods on organoid sensitivity and correlation with patient response.

## Data Availability

The datasets generated during and/or analysed during the current study are available from the corresponding author on reasonable request after ethical approval. The underlying code for this study is not publicly available but may be made available to qualified researchers on reasonable request from the corresponding author. The organoid models are available and can be requested via Foundation Hubrecht Organoid Biobank (https://www.hubrechtorganoidbiobank.org/).

## Abbreviations

5-FU: 5-Fluorouracil
ATP: Adenosine triphosphate
AIC: Akaike information criterion
AUC: Area under the curve
CRC: Colorectal cancer
CI: Confidence interval
CT: Computed tomography
DMSO: Dimethylsulfoxide
DNA: Desoxyribonucleïnezuur
DRC: Drug response curve
EGF(R): Epidermal growth factor (receptor)
FOLFOX: 5-FU & oxaliplatin
FOLFIRI: 5-FU & irinotecan
GR: Growth rate
GR_50_: Concentration that gives 50% growth rate inhibition
GR_50_1/2: Concentration that gives 50% growth rate inhibition of the upper and lower part of the biphasic curve
GR_AUC_: Area under the non-fitted ‘curve’ of the raw GR values
GR_max_: Concentration that gives maximal growth rate inhibition
HR: Hazard ratio
mCRC: Metastatic colorectal cancer
NAC: N-acetyl cysteine
NGS: Next generation sequencing
pMMR: Proficient mismatch repair
PDO: Patient-derived organoid
PFS: Progression-free survival
RECIST: Response evaluation criteria in solid tumours
SNP: Single-nucleotide polymorphism
