# Supplementary material for: Organoids as a biomarker for personalized treatment in metastatic colorectal cancer: drug screen optimization and correlation with patient response
Source: J Exp Clin Cancer Res. 2024 Feb 27;43:61. doi: 10.1186/s13046-024-02980-6 (PMC10898042; doi:10.1186/s13046-024-02980-6)
Supplement: Supplementary file 1 — Additional file 1: Supplementary Table 1. Composition of organoid culture medium. Supplementary Table 2. Chemotherapies and targeted treatments used in drug screens. Supplementary Table 3. Baseline characteristics of the cohort of patients. Supplementary Table 4. Quality control analysis of the drug screens showing the Z’-factor. Supplementary Fig. 1. Quality control analysis of the drug screens illustrating the difference between duplicate assays. Supplementary Fig. 2. Individual drug response curves for each PDO per treatment. Supplementary Fig. 3. Comparing different drug screening methods. Supplementary Fig. 4. The impact of different drug screening methods on organoid sensitivity and correlation with patient response. [file 13046_2024_2980_MOESM1_ESM.zip › Supplementary files/Additional file 2.docx]

Additional file 2

**Supplementary Table 2. Chemotherapies and targeted treatments used in drug screens**.

| Drug | Readout | Source  (catalogue number) | Target | Concentration range (μM) |
| --- | --- | --- | --- | --- |
| **5-FU** | CellTiter-Glo & CyQUANT | 15596885 (UMCU) | DNA synthesis | 0.01 – 2700 |
| **Oxaliplatin** | CellTiter-Glo & CyQUANT | 15532585 (UMCU) | DNA synthesis | 0.01 – 270 (CellTiter-Glo)  0.03 – 270 (CyQUANT) |
| **SN-38**  **(irinotecan)** | CellTiter-Glo & CyQUANT | Selleck Chemicals GmbH (S4908) | Topoisomerase I | 0.00001 – 1.8 |
| **5-FU & oxaliplatin (0.05)** | CellTiter-Glo | 15596885 (UMCU) + 15532585 (UMCU) | DNA synthesis | Anchor oxaliplatin: 0.05  5-FU: 0.01 – 2700 |
| **5-FU & oxaliplatin ratio 1.8:1** | CellTiter-Glo & CyQUANT | 15596885 (UMCU) + 15532585 (UMCU) | DNA synthesis | 5-FU: 0.0457 – 900  Oxaliplatin: 0.0254 – 500 |
| **5-FU + SN-38 (****0.01)** | CellTiter-Glo & CyQUANT | 15596885 (UMCU) + Selleck Chemicals GmbH (S4908) | DNA synthesis +  Topoisomerase I | Anchor SN-38: 0.01  5-FU: 0.01 – 2700 |
| **5-FU + SN-38 ratio 1500:1** | CyQUANT | 15596885 (UMCU) + Selleck Chemicals GmbH (S4908) | DNA synthesis +  Topoisomerase I | SN-38: 6.87 × 10^−6^ – 1.8  5-FU: 0.01 – 2700 |
| **Panitumumab** | CellTiter-Glo & CyQUANT | 15343561 (UMCU) | EGFR | 0.11 – 30,000 |
| **Staurosporine** | CellTiter-Glo & CyQUANT | Merck Life Science N. V. (37095) | Protein kinase inhibitor | 2 |
| **DMSO** | CellTiter-Glo & CyQUANT | VWR (ICNA0219605525) | Solvent | 0.2 |
| **PBS** | CellTiter-Glo & CyQUANT | Thermo Fisher Scientific (14190-144) | Solvent | 5 |

The compounds used in the organoid drug screens is described. Concentration ranges were optimized to ensure a full DRC would be obtained.

*Abbreviations*: 5-FU (5-fluorouracil), DMSO (dimethyl sulfoxide), DRC (drug response curve), EGFR (epidermal growth factor receptor), PBS (phosphate buffered saline), SN-38 (active metabolite of irinotecan), μM (micromolar), UMCU (University Medical Center Utrecht).
