# Supplementary material for: Organoids as a biomarker for personalized treatment in metastatic colorectal cancer: drug screen optimization and correlation with patient response
Source: J Exp Clin Cancer Res. 2024 Feb 27;43:61. doi: 10.1186/s13046-024-02980-6 (PMC10898042; doi:10.1186/s13046-024-02980-6)
Supplement: Supplementary file 1 — Additional file 1: Supplementary Table 1. Composition of organoid culture medium. Supplementary Table 2. Chemotherapies and targeted treatments used in drug screens. Supplementary Table 3. Baseline characteristics of the cohort of patients. Supplementary Table 4. Quality control analysis of the drug screens showing the Z’-factor. Supplementary Fig. 1. Quality control analysis of the drug screens illustrating the difference between duplicate assays. Supplementary Fig. 2. Individual drug response curves for each PDO per treatment. Supplementary Fig. 3. Comparing different drug screening methods. Supplementary Fig. 4. The impact of different drug screening methods on organoid sensitivity and correlation with patient response. [file 13046_2024_2980_MOESM1_ESM.zip › Supplementary files/Additional file 4.docx]

Additional file 4

**Supplementary Table 4. Quality control analysis of the drug screens showing the Z’-factor.**

|  | **Z’ -factor** | | |
| --- | --- | --- | --- |
| **Organoid ID** | **DMSO** | **PBS** | **PBS-DMSO** |
| 01 | 0,63 | 0,6 | 0,55 |
| 02 | 0,63 | 0,68 | 0,58 |
| 03 | 0,63 | 0,68 | 0,69 |
| 04 | 0,66 | 0,65 | 0,57 |
| 05 | 0,6 | 0,68 | 0,71 |
| ­06 | 0,59 | 0,62 | 0,57 |
| 07 | 0,43 | 0,74 | 0,71 |
| 08 | 0,74 | 0,7 | 0,68 |
| 09 | 0,7 | 0,73 | 0,48 |
| 10 | 0,66 | 0,7 | 0,69 |
| 11 | 0,6 | 0,56 | 0,59 |
| 12 | 0,67 | 0,73 | 0,75 |
| 13 | 0,6 | 0,58 | 0,57 |
| 14 | 0,68 | 0,69 | 0,74 |
| 15 | 0,65 | 0,78 | 0,75 |
| 16 | 0,55 | 0,64 | 0,64 |
| 17 | 0,73 | 0,73 | 0,71 |
| 18 | 0,62 | 0,67 | 0,59 |
| 19 | 0,72 | 0,67 | 0,68 |
| 20 | 0,55 | 0,54 | 0,57 |
| 21 | 0,67 | 0,75 | 0,69 |
| 22 | 0,76 | 0,73 | 0,68 |
| 23 | 0,72 | 0,72 | 0,73 |

The mean Z’-factor is shown for different compounds used as the negative control, per organoid line. *Abbreviations:* DMSO (dimethyl sulfoxide), PBS (phosphate buffered saline).
